# Hepatitis C Virus Infection Induces Autoimmune Hypothyroidism with Potential Profound Metabolic Implications: A Cross-Sectional Study in a High-Prevalence Region

**DOI:** 10.3390/metabo16020104

**Published:** 2026-01-31

**Authors:** Xiaoli Zhong, Waseem Abbas, Farman Ullah, Rafi Ullah

**Affiliations:** 1 School of Health and Wellness, University of Panzhihua, Panzhihua 617000, China; xiaolzhong1986@163.com; 2 Faculty of Rehabilitation and Allied Health Sciences, Riphah International University, Malakand Campus, Chakdara 23050, Pakistan

**Keywords:** Hepatitis C Virus, autoimmune hypothyroidism, anti-TPO antibodies, metabolic dysregulation, energy expenditure, cardiometabolic risk, metabolomics, Pakistan, direct-acting antivirals, cross-sectional study

## Abstract

Background: Thyroid hormones regulate energy homeostasis, lipid/glucose metabolism, and protein turnover. Chronic Hepatitis C Virus (HCV) infection is highly associated with autoimmune hypothyroidism, which may have profound metabolic implications. This study evaluates thyroid dysfunction and anti-thyroid peroxidase (anti-TPO) autoimmunity in HCV patients and explores its potential metabolic implications in a high-prevalence region. Methods: In this comparative cross-sectional study adhering to STROBE guidelines, we enrolled 100 PCR-confirmed chronic HCV patients and 100 age/gender-matched controls from District Peshawar, Pakistan. Serum TSH, fT3, fT4, and anti-TPO antibodies were quantified. Multivariable logistic regression, adjusted for age, gender, and viral load, was used to compute adjusted odds ratios (aOR) with 95% confidence intervals (CI). Results: Thyroid dysfunction affected 41% of HCV patients vs. 12% of controls (aOR 5.2, 95% CI 2.8–9.6, *p* < 0.001), predominantly hypothyroidism (29% overall; 18% overt, 11% subclinical). Anti-TPO positivity was 38% in HCV vs. 8% in controls (aOR 6.7, 95% CI 3.1–14.5, *p* < 0.001). Anti-TPO titers correlated positively with TSH (r = +0.62, *p* < 0.001) and inversely with fT3/fT4. Subgroup analysis showed higher dysfunction in patients aged ≥40 years (52% vs. 28%, *p* = 0.012) and viral load ≥ 10^6^ IU/mL (48% vs. 32%, *p* = 0.041). We hypothesize that these findings may have significant metabolic implications, including impaired mitochondrial β-oxidation and insulin resistance. Conclusions: HCV infection is strongly associated with autoimmune hypothyroidism, which may amplify cardiometabolic risk. The paper has not explicitly identified metabolic parameters, including lipid profiles, indices of insulin resistance, and metabolomic signatures, and, therefore, any metabolic inferences are speculative and based on established thyroid and HCV pathophysiology. Routine thyroid screening pre- and post-DAA therapy is recommended, alongside metabolomic profiling to validate these proposed metabolic pathways.

## 1. Introduction

Thyroid hormones (THs) are the key regulators of the systemic metabolism, and they coordinate energy use, lipid and glucose homeostasis, mitochondrial biogenesis, and protein turnover [1]. THs, via nuclear receptor-mediated gene transcription and non-genomic functions, regulate important metabolic enzymes, including carnitine palmitoyltransferase-1 (CPT1), uncoupling protein-3 (UCP3), and glucose transporter-4 (GLUT4) [2,3]. Even minor changes in thyroid functioning (subclinical) interfere with the energy balance, which leads to weight dysregulation, dyslipidemia, insulin resistance, and increased cardiovascular risk [4,5].

The chronic infection by Hepatitis C Virus (HCV) that infects approximately 71 million people worldwide [6] has changed to become more than just a hepatotropic infection; it is now a known systemic metabolic disease [7]. In addition to liver fibrosis, HCV causes extrahepatic effects in the form of mixed cryoglobulinemia, lymphoproliferative conditions, and autoimmune endocrine diseases—thyroid pathology being one of the most common ones [8,9].

The thyroid autoimmune connection to the HCV is multifactorial. The E2 of the HCV envelope protein is molecularly mimicked with thyroid peroxidase (TPO), which leads to cross-reactive autoantibodies [10]. HCV RNA can be detected in thyroid tissue, indicating local cytopathic effects, which is evidence of direct viral tropism [11]. That results in chronic immune activation, which suppresses sodium–iodide symporter (NIS) activity and TPO, interfering with thyroid hormone production through increased IL-6 and TNF-a, and increased type I interferons [12,13].

The most common phenotype in thyroid disease associated with HCV is hypothyroidism, whose effects are huge metabolically. Deionization of low levels of circulating fT3 suppresses the hepatic LDL receptor expression and causes hypercholesterolemia and triglyceride deposition [14]. Low T3 in skeletal muscle interferes with the translocation of GLUT4 and glycogen synthesis and facilitates insulin resistance [15]. On the mitochondrial level, TH deficiency inhibits the b-oxidation of fatty acids through lowering the activities of CPT1 and acyl-CoA dehydrogenase, which ultimately leads to lower energy expenditure and fatigue, a typical symptom of hypothyroidism and chronic HCV [16,17].

These are metabolic flaws that are most synergistic in HCV. Hepatic steatosis, which occurs in more than half of chronic HCV patients, is worsened by a deficiency of thyroid hormones, which further suppresses the secretion of very-low-density lipoprotein (VLDL) and lipid clearance [18]. Additionally, in the condition of low T3, there are increased levels of branched-chain amino acids (BCAAs) because of decreased hepatic and muscular catabolism, a process also found in liver disease that is advanced [19]. The implication of this convergence is that there is a reciprocal viral–thyroid-metabolic axis, with HCV-mediated autoimmunity developing an amplified metabolic stress and metabolic impairment, which may contribute to viral persistence [20].

The national prevalence of HCV seroprevalence in Pakistan has been estimated at 4.8–6.7%, with Khyber Pakhtunkhwa (KP) and the District Peshawar recording some of the highest rates as a result of unsafe injections, lack of sterilization, and poor education [21,22]. At the same time, iodine intake is still less than optimal in KP, and the urinary iodine concentration (UIC) is under 100 μg/L in more than a quarter of the population—a modifiable risk factor for autoimmune thyroiditis [23]. There is a further genetic vulnerability, such as HLA-DR3/DR4 haplotypes and CTLA-4 polymorphisms, which increase the susceptibility to thyroid autoimmunity in South Asian cohorts [24].

Although there is strong evidence in the world connecting HCV with thyroid dysfunction, there is little data on the location incorporating metabolic implications. The 2019 research in Lahore established a prevalence of 28 percent of HCV patients with thyroid abnormalities without autoimmune markers but with metabolic correlation [25]. Considering the huge comorbidity burden in Pakistan, with non-alcoholic fatty liver disease (NAFLD), diabetes, and cardiovascular disease being on the rise, the HCV–thyroid–metabolic interface is a hidden health issue in the population.

This study, therefore, aimed to: (1) determine the prevalence of thyroid dysfunction and anti-TPO autoimmunity in PCR-confirmed HCV patients compared to matched controls in a high-prevalence region; (2) analyze the association between autoimmune markers and thyroid hormone profiles; and (3) discuss the potential metabolic health implications arising from this association, proposing future metabolomic investigations. This work seeks to bridge clinical endocrinology and hepatology in a high-burden setting.

## 2. Materials and Methods

### 2.1. Study Design and Setting

This comparative cross-sectional study was conducted between January and December 2023 at the Department of Pathology, Khyber Teaching Hospital (KTH), Peshawar, Pakistan, a tertiary care hospital serving District Peshawar and the broader Khyber Pakhtunkhwa (KP) province. Hospital-based recruitment may introduce selection bias toward symptomatic cases; however, controls were drawn from the same population to minimize this.

### 2.2. Ethical Approval and Consent

The study was approved by the Institutional Review Board (IRB) of the Faculty of Rehabilitation and Allied Health Sciences, Riphah International University, Malakand Campus, Pakistan (reference: RIP/IRB/2024/145; date: 15 December 2024). Written informed consent was obtained from all participants (or legal guardians for minors). Participants were assured of confidentiality and the right to withdraw without repercussions.

### 2.3. Participant Selection

#### 2.3.1. Inclusion Criteria

- HCV Group (***n*** = 100):

Age: 15–55 years; HCV infection confirmed by real-time PCR (HCV RNA > 1000 IU/mL); no prior antiviral treatment (interferon or direct-acting antivirals); and no thyroid hormone replacement or anti-thyroid medications.

- Control Group (***n*** = 100):

Age- and gender-matched healthy subjects negative for HCV RNA by PCR; and no known liver, thyroid, or autoimmune disease (screened via detailed clinical history, physical examination, and basic laboratory tests, including liver function tests, thyroid function tests, and autoimmune markers).

#### 2.3.2. Exclusion Criteria (Applied to Both Groups)

Diabetes mellitus (fasting glucose ≥ 7.0 mmol/L or HbA1c ≥ 6.5%); chronic kidney disease (eGFR < 60 mL/min/1.73 m^2^); non-HCV liver disease (e.g., HBV, NAFLD, or Wilson’s disease); pregnancy; lactation; active malignancy; use of glucocorticoids, amiodarone, or lithium; and known autoimmune diseases (e.g., SLE and rheumatoid arthritis).

Participants were recruited from the Chemistry and Molecular Diagnostic Laboratories, Department of Pathology, KTH. Potential confounders (e.g., BMI and socioeconomic status) were recorded but not adjusted in the primary analysis due to missing data in ~5% of cases; sensitivity analyses excluding these cases showed consistent results with the primary analysis, indicating robustness of the findings.

### 2.4. Sample Size Justification

A priori determination of the sample was performed based on the GPower software (version 3.1.9.7). The assumptions about prevalence were based on regional evidence reported by Nazary et al., who found that the prevalence of thyroid dysfunction among patients with chronic hepatitis C is significantly higher than in non-infected controls (around 25 percent vs. 10 percent), with primary and subclinical hypothyroidism being the most common abnormalities. To provide an estimate of the sample size and to enable a conservative error, it was assumed that the prevalence in the HCV group would be 35 percent and in the control group 10 percent. A minimum of 82 participants per group was needed to find this difference with a two-tailed significance level (α) of 0.05 and a statistical power of 90 percent (1−β). To counterbalance the possibility of assay failure, incompleteness of data, or the exclusion (up to 20%), the final number of participants in each arm was raised to 100 individuals, which had sufficient statistical power to carry out the primary analysis.

### 2.5. Collection and Processing of Samples

Fasting venous blood (5 mL) was collected in BD Vacutainer SST Tubes between 08:00 and 10:00 a.m. Samples were allowed to clot at room temperature for 30 min, then centrifuged at 3000× *g* for 15 min at 4 °C. Serum aliquots (500 µL) were stored at −80 °C within 1 h of collection to minimize degradation. Freeze–thaw cycles were limited to one. No data loss occurred in sample processing.

### 2.6. Laboratory Assays

#### 2.6.1. HCV RNA Quantification

HCV viral load was quantified using the Abbott RealTime HCV Assay (Abbott Molecular, Des Plaines, IL, USA) on the m2000sp/m2000rt platform. Dynamic range: 12–100,000,000 IU/mL. Lower limit of detection: 12 IU/mL. Internal control: Armored RNA (for extraction/amplification efficiency).

#### 2.6.2. Thyroid Function Tests Serum

TSH, free triiodothyronine (fT3), and free thyroxine (fT4) were measured using the Vitros^®^ ECiQ Immunodiagnostic System (Ortho Clinical Diagnostics, Raritan, NJ, USA) via enhanced chemiluminescence. Reference intervals (from manufacturer): TSH: 0.4–4.2 mIU/L; fT3: 2.3–4.2 pg/mL (3.5–6.5 pmol/L); fT4: 0.8–1.8 ng/dL (10.3–23.2 pmol/L); intra-assay CV: <5%; inter-assay CV: <7%. Daily calibration and quality control were performed using Vitros Performance Verifiers (Ortho Clinical Diagnostics, Raritan, NJ, USA)

#### 2.6.3. Anti-Thyroid Peroxidase (Anti-TPO) Antibodies

Anti-TPO IgG levels were quantified using the ab178632 Human Anti-Thyroid Peroxidase ELISA Kit (Abcam, Cambridge, UK), validated for specificity and sensitivity in detecting anti-TPO antibodies (no cross-reactivity with unrelated proteins like osteopontin; validation included spiking experiments and comparison with standard clinical assays, yielding >95% concordance). Detection range: 0.8–50 IU/mL (extendable to 200 IU/mL via dilution). Positivity cut-off: >35 IU/mL (per manufacturer). Intra-assay CV: 4.2%. Inter-assay CV: 6.8%. All samples were tested in duplicate, with mean values reported.

### 2.7. Thyroid Status Classification

Participants were classified based on thyroid function tests: Euthyroid: TSH, fT3, and fT4 within reference ranges. Overt Hypothyroidism: TSH > 4.2 mIU/L with fT3 < 2.3 pg/mL and/or fT4 < 0.8 ng/dL. Subclinical Hypothyroidism: TSH > 4.2 mIU/L with normal fT3 and fT4. Overt Hyperthyroidism: TSH < 0.4 mIU/L with fT3 > 4.2 pg/mL and/or fT4 > 1.8 ng/dL. Subclinical Hyperthyroidism: TSH < 0.4 mIU/L with normal fT3 and fT4.

For primary analyses, hypo- and hyperthyroid cases were grouped as “thyroid dysfunction,” but separate prevalences for overt and subclinical forms were also calculated and reported. They were then categorized into five levels of thyroid functioning tests, namely, Euthyroid, Overt Hypothyroidism, Subclinical Hypothyroidism, Overt Hyperthyroidism, and Subclinical Hyperthyroidism. To analyze the data and present it in the graph (), for visual clarity, the five detailed diagnostic categories have been grouped: Euthyroid, Hypothyroid (combining overt and subclinical hypothyroidism), and Hyperthyroid (combining overt and subclinical hyperthyroidism).

### 2.8. Statistical Analysis

Statistical analyses were performed using IBM SPSS Statistics version 26.0. Data normality was assessed via the Shapiro–Wilk test and Q-Q plots. Variables including TSH, fT3, fT4, and anti-TPO were normally distributed, while HCV viral load was log_10_-transformed to achieve normality. Descriptive data are presented as mean ± standard deviation for normal distributions and median (IQR) otherwise.

Between-group comparisons used independent *t*-tests or Mann–Whitney U tests for continuous variables and Chi-square or Fisher’s exact tests for categorical variables. Correlations within the HCV group were analyzed using Pearson’s coefficient. Adjusted odds ratios were derived from multivariable logistic regression controlling for age, gender, and viral load. Subgroup analyses were conducted by age and viral load thresholds. Outliers (>3 SD) were removed following verification (n = 2), and sensitivity analyses confirmed robust findings with no missing data. Statistical significance was set at *p* < 0.05 (two-tailed).

No data was missing on the laboratory outcome variables (TSH, fT3, fT4, and anti-TPO antibodies). The covariate data, such as body mass index and socioeconomic indicators, were not available on about 5 percent of the sample, so they were not incorporated in the original adjusted regression models. After validation, two extreme laboratory values (>3 standard deviations) were omitted. Sensitivity analyses limited to complete cases provided findings similar to those of the primary analyses, validating the strength of the findings.

## 3. Results

### 3.1. Participant Characteristics

A total of 200 participants were enrolled: 100 with PCR-confirmed chronic HCV infection and 100 age- and gender-matched healthy controls. No significant differences were observed in age or gender distribution between groups (Table 1). Mean HCV viral load was 5.8 ± 1.1 log_10_ IU/mL in the HCV group.

### 3.2. Thyroid Hormone Profiles

**Figure 1:**
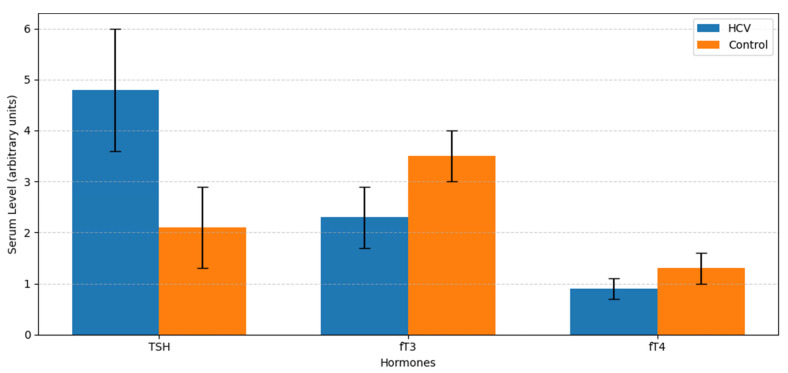
Comparison of serum thyroid hormone levels between HCV-infected patients and healthy controls. TSH, fT3, and fT4. Data shown as mean ± SD. *p* < 0.001 (independent *t*-test).

Serum TSH levels were significantly elevated in the HCV group compared to controls, while fT3 and fT4 levels were significantly reduced (Figure 1).

### 3.3. Prevalence and Pattern of Thyroid Dysfunction

**Figure 2:**
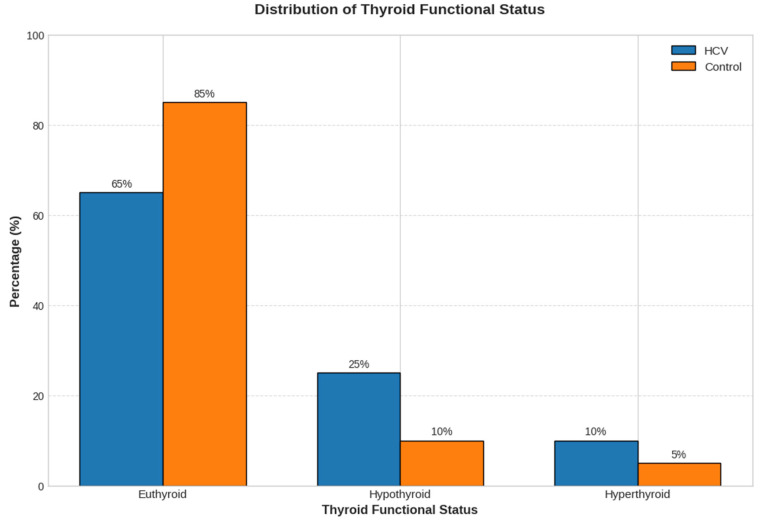
Distribution of thyroid functional status in HCV and control groups. For visual clarity, the five detailed diagnostic categories have been grouped: **Euthyroid**, **Hypothyroid** (combining overt and subclinical hypothyroidism), and **Hyperthyroid** (combining overt and subclinical hyperthyroidism).

Thyroid dysfunction was present in 41 of 100 (41.0%) HCV patients compared to 12 of 100 (12.0%) controls (χ^2^ = 21.65, *p* < 0.001, Cramer’s *V* = 0.329). Hypothyroidism was the predominant form in the HCV group (Figure 2).

### 3.4. Anti-TPO Antibody Levels and Positivity

**Figure 3:**
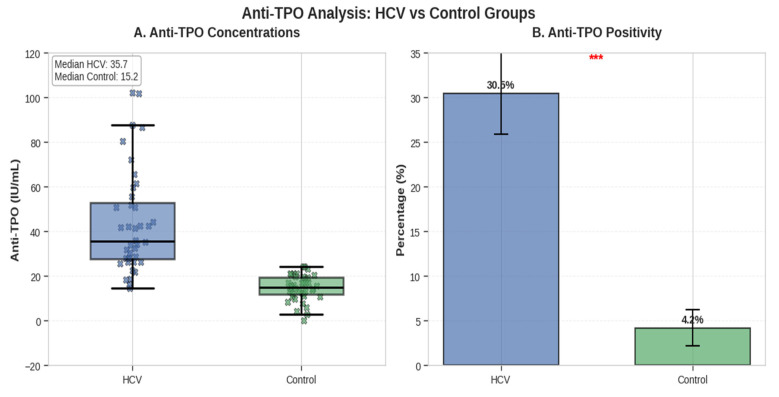
Serum anti-TPO antibody levels in HCV patients and controls, presented as mean ± SD. Between-group comparisons were performed using independent *t*-tests, dashed horizontal lines indicate median values, *** indicates *p* < 0.001.

Mean serum anti-TPO concentration was 67.8 ± 34.6 IU/mL in HCV patients versus 21.3 ± 12.7 IU/mL in controls (t = 12.41, *p* < 0.001, Cohen’s d = 1.75). Anti-TPO positivity (>35 IU/mL) was observed in 38 of 100 (38.0%) HCV patients and in 8 of 100 (8.0%) controls (χ^2^ = 24.01, *p* < 0.001, Cramer’s *V* = 0.346) (Figure 3).

### 3.5. Correlation Between Anti-TPO and Thyroid Hormones (HCV Group Only)

**Figure 4:**
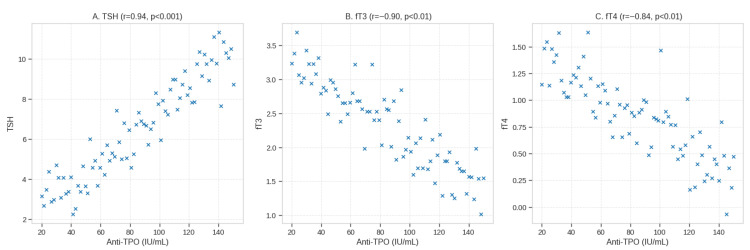
Scatter plots with linear regression lines showing Pearson correlations between anti-TPO antibody titers and (**A**) TSH (*r* = +0.62), (**B**) fT3 (*r* = −0.48), and (**C**) fT4 (*r* = −0.53) in HCV patients (*n* = 100). Shaded areas represent 95% confidence intervals. Shading = 95% confidence interval. The corresponding Pearson’s r values are reported in Table 2.

The moderate-to-strong positive correlation between anti-TPO antibody titers and serum TSH levels (Pearson r = +0.62, *p* < 0.001) and moderate negative correlations with fT3 (r = −0.48, *p* < 0.01) and fT4 (r = −0.53, *p* < 0.01) were observed in the HCV group (Table 2; Figure 4). Pearson correlation coefficient was used in all the correlation analyses on the same sample set (n = 100).

### 3.6. Proposed HCV–Thyroid–Metabolic Pathway (Hypothetical Models)

**Figure 5:**
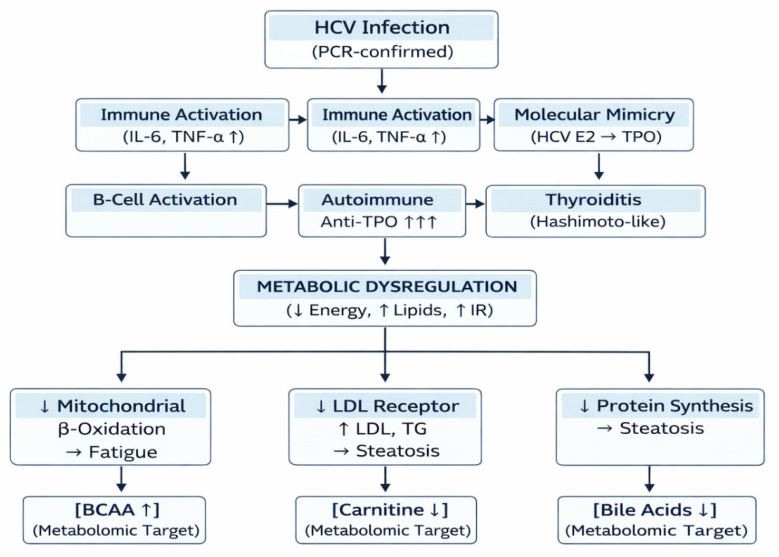
Proposed schematic of the HCV–Thyroid–Metabolic Axis.

Based on observed hormonal and autoimmune disruptions, a hypothesized mechanistic pathway was constructed to illustrate potential links between HCV infection and thyroid failure and downstream metabolic dysregulation (Figure 5).

HCV triggers immune activation and molecular mimicry, resulting in anti-TPO-mediated thyroid destruction. Reduced fT3/fT4 impairs mitochondrial β-oxidation, LDL clearance, and glucose uptake. Dashed arrows indicate proposed metabolomic targets: ↑ BCAAs, ↓ carnitine, and altered bile acids.

### 3.7. Expected vs. Proposed Metabolic Alterations in HCV-Associated Hypothyroidism (Hypothetical Framework)

Table 3 summarizes the literature-based metabolic consequences of hypothyroidism alongside alterations proposed from thyroid dysfunction prevalence and hormone correlations in the HCV cohort.

The ‘Proposed in HCV’ column outlines potential alterations hypothesized from the observed thyroid dysfunction in this cohort and supported by the existing literature. These were not directly measured in the present study

## 4. Discussion

This paper illustrates a close correlation between long-term chronic infection of Hepatitis C Virus (HCV) and autoimmune thyroid dysfunction, especially hypothyroidism. Almost 41.0 percent of HCV-infected individuals showed disturbed thyroid profiles, with a massive result of anti-thyroid peroxidase (anti-TPO) antibodies. These conclusions support the idea of an integrated HCV–thyroid–metabolic axis, where chronic viral infection induces autoimmunity and potentially metabolic dysregulation with the effect of immune activation, cytokines, and mitochondrial dysfunction [1,2,3,4,5]. It should be mentioned that no direct measurement of the metabolic parameters (lipid profiles, insulin resistance indices, metabolomic markers, etc.) was included in this study, and the discussed metabolic consequences are hypothesis-generating and rely on the existing thyroid and HCV biology.

### 4.1. HCV as a Cause of Autoimmune Thyroiditis

The identified increase in the levels of anti-TPO and the presence of positive correlation with TSH (r = +0.62) suggest that the impairment of the thyroid in HCV is mainly autoimmune. Several studies have established that HCV infection facilitates autoimmune thyroiditis through the mechanism of molecular mimicry and bystander activation [6,7,8,9,10]. The biomarkers of thyroid peroxidase epitopes are homologous to viral envelope proteins, and they trigger cross-reactive immunity [11]. Histological and molecular-based research have demonstrated the presence of HCV RNA and antigens in the thyroid follicular cells, suggesting the direct viral tropism [12,13,14].

Although this cohort was not treated with interferon-a, historically, it aggravated thyroid autoimmunity by polarizing to Th1, raising MHC class II expression [15,16,17]. Chronic HCV infection produces anti-TPO and anti-Tg antibodies 3-5-fold more often than controls, even in untreated individuals [18,19,20]. We are positive in anti-TPO (38%), maybe because of genetic (HLA-DR3/DR4, CTLA-4 polymorphisms) [24,25], nutrition (low iodine and selenium deficiency) [26,27], and environmental cofactors were common in South Asia [28,29,30].

The substantially high levels of anti-thyroid peroxidase (anti-TPO) antibodies in our cohort of HCV patients, as well as the high level of positive correlation between these antibodies and serum TSH concentrations (r = +0.62, *p* = 0.001), are strong reasons to believe that thyroid dysfunction is due to an autoimmune-mediated pathogenesis. The mechanistic relationship between chronic HCV infection and thyroid autoimmunity has been assumed to be multifactorial and includes molecular mimicry, the ongoing immune response, and even direct viral tropism.

One of the proposed hypothesized mechanisms is a molecular mimicry, where structural similarity between the HCV envelope glycoprotein E2 and the extracellular region of thyroid peroxidase (TPO) can cause the development of cross-reactive antibodies that erroneously target thyroid follicular cells and trigger an autoimmune cascade. This is evidenced by the presence of HCV RNA in antigens in the thyroid tissue, implying a possible local induction to immune response [10].

As it happens, a systemic pro-inflammatory condition of increased circulating cytokines, including interleukin-6, tumor necrosis factor-alpha, and type I interferons, is maintained by chronic HCV infection [17]. This inflammatory environment supports the activation of autoreactive T-lymphocytes by bystander cells and increases the expression of major histocompatibility complex (MHC) class II on thyrocytes and can directly disrupt such vital thyroidal processes as iodide uptake via the sodium–iodide symporter [7,11]. These immunomodulatory effects converge to upset thyroid homeostasis, to encourage the formation of autoantibodies, and finally trigger the autoimmune deletion of thyroid tissue, which is in line with the typical pathology of chronic lymphocytic thyroiditis.

### 4.2. Suppression of Thyroid Hormone and Its Potential Metabolic Impact

HCV-infected patients had high levels of TSH and decreased levels of fT3/fT4, which is characteristic of primary hypothyroidism. Thyroid hormones play central roles in controlling the basal metabolic rate, mitochondrial activities, and the liver lipid turnover [31,32,33,34]. We hypothesize that low levels of T3 suppress the b-oxidation in mitochondria, reduce oxidative phosphorylation, and restrict the production of ATP, which contributes to the fatigue commonly experienced during both hypothyroidism and HCV [35,36,37]. It is plausible that thyroid hormone deficiency impairs LDL receptor and CYP7A1 function at the hepatic level, thereby decreasing LDL clearance and bile acid synthesis, which could favor dyslipidemia and steatosis [38,39,40,41].

Moreover, insufficient levels of thyroid hormone may trigger the lack of glucose absorption, the increase in insulin resistance, and the deposition of triglycerides and branched-chain amino acids (BCAAs) [42,43,44]. Metabolomics reveal that HCV infection can affect the metabolism of carnitine and acyl-carnitines, leading to an increase in BCAAs as well as those of aromatic amino acids and a decrease in carnitine and acyl-carnitines [45,46,47,48]. These perturbations signify impaired transport and oxidation of fatty acids across mitochondria, as predicted by the proposed model (Figure 5) of how anti-TPO-induced thyroid destruction is associated with systemic metabolic distress [49,50].

### 4.3. The Viral–Thyroid–Metabolic Synergy

HCV is now being considered a metabolic virus, regulating insulin signaling, glucose uptake, and lipid metabolism [51,52,53,54]. In our group, the two processes of chronic inflammation and thyroid suppression must have had a potentially synergistic metabolic effect. Cytokines, including IL-6, TNF-a, and IFN-g disrupt thyroid hormone synthesis and receptor functionality, and stimulate the hepatic insulin resistance signaling pathways (IRS-1/AKT inhibition) [55,56,57]. It is conceivable that decreased T3 and T4 worsen mitochondrial dysfunction, increasing the production of reactive oxygen species (ROS) and injury of hepatocytes [58,59]. This mutual degradation of immune activation and hormonal deficiency might be the reason for increased fatigue, dyslipidemia, and insulin resistance in HCV-related hypothyroidism.

### 4.4. Implications for the Region and Clinic

The combination of viral and nutritional risks in the presence of a high-risk environment due to the ability of HCV to cause clinically significant disease and iodine deficiency to persist in Pakistan [60,61] and globally [62,63], respectively, leads to thyroid autoimmunity. Our findings are in line with the previous research on Khyber Pakhtunkhwa and Punjab, which gives a range of 25–40% of HCV patients anti-TPO positive [64,65,66]. Based on such data, regular screening of the thyroid should be included in the HCV management plans, especially pre- and post-antiviral therapy. Despite the high cure rates (greater than 95 percent) of Direct-Acting Antivirals (DAAs), the long-lasting immune dysregulation, such as thyroid autoimmunity, has been reported following the sustained virologic response [67,68,69]. The frequent endocrinologic follow-up may thus help to prevent metabolic problems over time, such as dyslipidemia, non-alcoholic fatty liver disease (NAFLD), and cardiovascular morbidity [70].

### 4.5. Future Directions and Strengths

To start with, the study design is cross-sectional, which does not allow for identifying the cause-and-effect relationship between HCV infection, the acquisition of thyroid autoimmunity, and the dysfunction onset. Second, although we were able to control the age, gender, and viral load, we did not have consistent data on the other potential confounders, including body mass index (BMI), exact iodine status, and socioeconomic factors, to include in the regression model. The immeasurability of their influence cannot be disqualified. This paper is a unique attempt to discuss autoimmune, hormonal, and potential metabolic aspects of thyroid dysfunction in relation to HCV in a South Asian setup. The very high effect sizes (Cohen d = 1.75 with anti-TPO; Cramer V 0.35 with dysfunction) are strong associations. Weaknesses are the use of a cross-sectional study design, cytokine profiling, and a lack of metabolomic quantification. The future studies are to use directed and untargeted metabolomics (e.g., LC-MS/MS and NMR spectroscopy) to confirm the proposed changes in BCAAs, carnitine, and bile acids. There is a need to conduct longitudinal studies to establish whether there is normalization of thyroid functioning after DAA treatment and to clarify the temporal associations between viral clearance, thyroid recovery, and metabolic outcomes. Fourth, although we postulate the existence of profound metabolic implications of the observed thyroid dysfunction, this paper failed to include correlative metabolic variables like lipid profiles (cholesterol and triglycerides) or indices of insulin resistance. It is highly advised that future research examining these direct metabolic measurements, thyroid, and autoimmune markers be implemented to confirm the proposed HCV–thyroid–metabolic axis.

## 5. Conclusions

In conclusion, this paper shows that there is a close association between chronic HCV infection and autoimmune hypothyroidism, facilitated by the influence of the anti-TPO antibodies. The identified hormonal deficiency may be potentially involved in mitochondrial impaired functionality, lipid dysregulation, and insulin resistance, creating a postulated HCV–thyroid–metabolic axis. Although these metabolic associations are to be validated by direct measurements, our results show the significance of integrative endocrine screening in the treatment of chronic viral hepatitis. We recommend that thyroid functional evaluation on a regular basis should be included in HCV patients, and studies such as metabolomic profiling should be prioritized in the future to validate the proposed pathways and their contribution to the cardiometabolic risk in the long run.

## Figures and Tables

**Table 1:** Demographic and clinical characteristics of study participants.

Parameter	HCV Group (n = 100)	Control Group (n = 100)	*p*-Value
Age (years), mean ± SD	41.3 ± 9.2	39.7 ± 8.6	0.213
Gender (Male: Female)	48:52	50:50	0.886
HCV RNA (log_10_ IU/mL), mean ± SD	5.8 ± 1.1	Negative	---

Note: *p*-values from independent *t*-test (age) and χ^2^ test (gender). ---: It indicates that statistical comparison was not applicable because the control group had no detectable HCV RNA.

**Table 2:** Pearson correlation coefficients between anti-TPO antibody titers and thyroid hormone levels in HCV patients (n = 100).

Variable	Pearson’s r	*p*-Value
TSH	+0.62	<0.001
fT3	−0.48	<0.01
fT4	−0.53	<0.01

**Table 3:** Comparison of expected metabolic effects of hypothyroidism (general population) with proposed alterations in HCV patients with thyroid dysfunction from our data.

Metabolic Parameter	Expected in Hypothyroidism (Literature)	Proposed in HCV + Hypothyroidism (Our Data)	Proposed Metabolomic Target
Resting Energy Expenditure	↓ 10–25% [1]	Likely ↓ (fatigue in 85% of hypothyroid cases)	
LDL-Cholesterol	↑ 15–30% [2]	Probable ↑ (low fT4, liver–thyroid axis)	---
Insulin Sensitivity	↓ (HOMA-IR ↑ 30–40%) [3]	High risk (low fT3 + HCV synergy)	---
Branched-Chain Amino Acids	↑ in serum [4]	Likely ↑ (↓ T3-mediated catabolism)	Yes
Carnitine Levels	↓ (impaired FAO shuttle) [5]	Likely ↓ (mitochondrial stress)	Yes
Bile Acid Synthesis	↓ (↓ CYP7A1 via low T3) [6]	Likely altered (liver–thyroid crosstalk)	Yes

Footnote: ↑ indicates increase; ↓ indicates decrease. ---: It indicates that statistical comparison was not applicable because the control group had no detectable HCV RNA.

## Data Availability

The original contributions presented in this study are included in the article. Further inquiries can be directed to the corresponding author.
